# Mammary nodules as incidental findings on chest computed tomography: a retrospective analysis on their frequency and predictive value

**DOI:** 10.1007/s11547-023-01670-1

**Published:** 2023-07-04

**Authors:** Maria Francesca Agliata, Naomi Calabrò, Stefano Tricca, Anna Maria Rampi, Anna Clelia Lucia Gambaro, Daniela Ferrante, Alessandro Carriero

**Affiliations:** 1 SCDU Radiodiagnostica, Ospedale Maggiore della Carità, Novara, Italy; 2 Dipartimento di Medicina Traslazionale, Università del Piemonte Orientale, Novara, Italy

**Keywords:** Breast cancer, CT scan, Mammography, Incidental findings

## Abstract

**Purpose:**

To evaluate the frequency of mammary nodules as incidental findings on chest CT scans and to determine a correlation between semiological features and mammographic and histopathological outcomes.

**Methods:**

A total of 42,864 chest CT scans performed on patients with breast-unrelated working diagnoses by the Radiology Department at AOU Maggiore della Carità, between 1st January 2016 and 30th April 2022, were analysed. Sixty-eight patients (3 males and 65 females) with mammary nodule CT detection were selected and subjected to mammography, mammary ultrasound and, eventually, biopsy.

**Results:**

Thirty-five of the 68 patients received a histopathological confirmation of malignancy. According to Pearson’s Chi-square test, the CT features most likely associated with BI-RADS 5 following mammography were post-contrast enhancement (*p* = 0.001), margin irregularity (*p* = 0.0001), nipple retraction (*p* = 0.001), skin thickening (*p* = 0.024), and the presence of structurally atypical lymph nodes suspicious for metastatic involvement (*p* = 0.0001). The CT features predictive of a biopsy positive for malignancy were post-contrast enhancement (*p* = 0.0001), margin irregularity (*p* = 0.0001), and the presence of suspicious lymph nodes (*p* = 0.011). Finally, 63.4% of patients with a working diagnosis related to cancer were diagnosed with breast cancer.

**Conclusion:**

Chest CT incidental findings of mammary nodules had a 0.21% incidence rate. The accurate description of some CT scan features, such as post-contrast enhancement, margin irregularity, nipple retraction, skin thickening and the presence of structurally atypical lymph nodes, may help to establish a radiological suspicion of malignancy, especially if these characteristics are supported by a working diagnosis of cancer.

## Introduction

Breast cancer is the most common malignancy among females [1, 2]. The ever-growing use of CT imaging as the primary diagnostic tool for thoracic pathologies [3] has led to a significant increase in incidental findings of mammary nodules. Thus, it is essential that radiologists know how to recognise specific features of the latter [4].

Even though mammary tumours still represent a minority of the incidentalomas requiring further investigation (0.6–1%), statistically significant incidence rates of breast cancer have been reported in patients subsequently directed to mammography, ranging between 17 and 36% [5–7]. These nodules include primary or secondary malignancies, as well as benign tumours such as cysts, fibroadenomas, gland thickening areas, lipomas, and dystrophic calcifications [8, 9].

Previous studies have reported a malignancy incidence rate of 0.3% among nodules detected by basal CT and of 0.6% among those identified through organic iodinated contrast media on CT imaging [10, 11]. However, CT exposes breast tissues to higher doses of radiation compared to standard mammography (4–7 mSv vs 0.2–0.3 mSv) [12].

This study aims to determine the frequency and the tomographic features of mammary nodules detected as incidental findings on chest CT scans.

## Materials and methods

### Ethics and study design

This study was a retrospective analysis aimed to evaluate the frequency and the tomographic features of mammary nodules detected as incidental findings on chest CT. It was conducted in compliance with the current norms of good clinical practise (GCP) and respecting the patient anonymity and rights to privacy. The study is approved by the Department of Translational Medicine University of the Eastern Piedmont (Prot. n. 0003609). The analysis collected data from 42,864 chest CT scans performed by the Radiology Department at the AOU Maggiore della Carità in either elective or urgency setting between 1st January 2016 and 30th April 2022. The CT scans included in the study were performed under basal conditions or using an organic iodinated contrast agent and had to include the mammary glands in their field of vision (FOV).

### Study population

Out of 42,864 chest CT scans analysed, 158 patients were diagnosed with at least one incidental mammary nodule: 68 (43%) underwent a subsequent diagnostic follow-up at our Radiology Department.

Inclusion criteria were as follows: (i) the availability of a CT scan (with organic iodinated contrast medium or not) either in election or urgency; (ii) the detection and description of an incidental mammary nodule, mammary tumour, or contrast-enhanced area in the breast tissues; and (iii) the patient’s radiological follow-up through either breast clinical examination, mammography, mammary ultrasound, or biopsy.

Exclusion criteria were as follows: (i) patients who had already undergone chest CT as a diagnostic or follow-up examination for a known breast malignancy; (ii) patients who continued their follow-up at another centre; and (iii) CT scans which did not include the whole breast parenchyma in the study volume.

### Image acquisitions and analysis

The radiological data were collected through the following imaging equipment: CT scan (Philips 256, 128 and 32—slices; Ultravist 370 mg/ml and Iomeron 400 mg/ml as organic iodinated contrast agents), mammography (Hologic Selenia Dimension and Siemens Mammomat Inspiration 3D), and ultrasound (Philips).

To characterise CT-detected mammary incidentalomas, each radiologist had to fill in a forced-choice module inclusive of size, foci (multifocal, multicentric, bilateral), contrast-enhancement, margins (regular or not), the presence of microcalcification, nipple retraction, skin thickening, or structurally atypical lymph nodes suspicious for metastatic involvement (short axis > 1 cm, rounded in shape, loss of the fatty hilum). The incidental finding of a breast malignancy was then correlated with the working diagnosis, focusing on the distinction between oncological and non-oncological cases. The selected patients were subsequently visited by a breast radiologist and directed to a mammography and ultrasound investigation. All cases suspicious for breast malignancy (BI-RADS 4-5) also underwent histological assessment through breast biopsy.

All radiological data were digitally stored in the hospital Picture Archiving and Communication System (PACS), accessible only to authorised medical staff through personalised username and password.

### Statistical analysis

The patients’ demographics and clinical features were summarised through descriptive statistics. Specifically, absolute and percentage frequencies were used as qualitative variables. As for quantitative variables with a Gaussian distribution, arithmetic means and standard deviations were applied, while in the case of non-Gaussian distribution, medians and interquartile ranges were used. The suspected association between CT-detected lesions and malignancy was assessed through logistic regression. The OR and its relative confidence interval were set at 95%. Statistical significance was considered reached at a *p* value < 0.05. Statistical analyses were performed by STATA v17.

## Results

### Study cohort

Between 1st January 2016 and 30th April 2022, 42,864 chest CT scans with mammary glands in the FOV were performed by the Radiology Department at the AOU Maggiore della Carità, in either elective or urgency setting. Incidental focal mammary lesions were detected in 158 cases (0.37%) and described in the CT reports. Among these patients, 22 were excluded because they chose to continue their radiological assessment at a different centre, while other 68 because of a current or past history of breast cancer for which they were undergoing staging and re-staging examinations, respectively. Excluding these 68 patients affected by a breast malignancy, the incidental detection of mammary nodules by CT during the study period was 0.21%.

A total of 68 patients were selected to evaluate the correlation between the CT incidental findings and the actual clinical and histopathological diagnosis. Of them, 65 (95.6%) were females and 3 (4.4%) males, with a mean age at diagnosis of 72.3 years (range 62–82). The CT incidentalomas were detected through basal examination in 16 (23.5%) cases, while 52 (76.5%) had undergone CT with iodinated contrast medium. The patients were grouped in two categories based on their working diagnosis: non-oncological [38 patients (55.8%), 22 (57.9%) with a benign nodule and 16 (42.1%) with a malignant one] and oncological [30 (44.2%) patients, 11 (36.6%) with a benign nodule and 19 (63.4%) with a malignant one] (Table 1).

**Table 1:** Correlation between sample size, clinical question and histopathological results

Clinical question	No. of patients	Benign mammary nodule	Malignant mammary nodule
Non-oncological	38	22	16
Oncological	30	11	19

### CT characteristics of the nodules

The mean diameter of the CT-detected tumours was 20.4 mm (range 5–80 mm). Out of the 68 selected CT scans, unifocal lesions were identified in 46 (67.7%) cases, while the other 22 (32.3%) were characterised by multifocality and/or multicentricity. The nodule margins were regular in 32 (47%) patients and irregular in the remaining 36 (53%). Intranodular microcalcifications were identified in only 9 (13%) cases, nipple retractions in 12 (18%), and skin thickening in 14 (21%) patients. In 19 (28%) patients, the locoregional lymph nodes were deemed suspicious for metastatic involvement.

### Mammography and ultrasound analysis

During the patients’ follow-up, the mammary incidentalomas were assessed by mammography and/or ultrasound examination. In both cases, the lesions were classified according to the BI-RADS scale. As for mammograms, the findings were compatible with *B*1 in 9 (13.24%) cases, *B*2 in 17 (25%), *B*3 in 7 (10.29%), *B*4 in 6 (8.82%), and *B*5 in 20 (29.41%). As for the ultrasound examination, 2 patients were classified as *B*1 (2.94%), 22 as *B*2 (32.35%), 8 as *B*3 (11.76%), 8 as *B*4 (11.76%), and 26 as *B*5 (38.24%). Out of the 68 patients included in the analysis, 2 (2.94%) and 9 (13.24%) exclusively underwent mammography or ultrasound.

### Histology findings

**Fig. 1:**
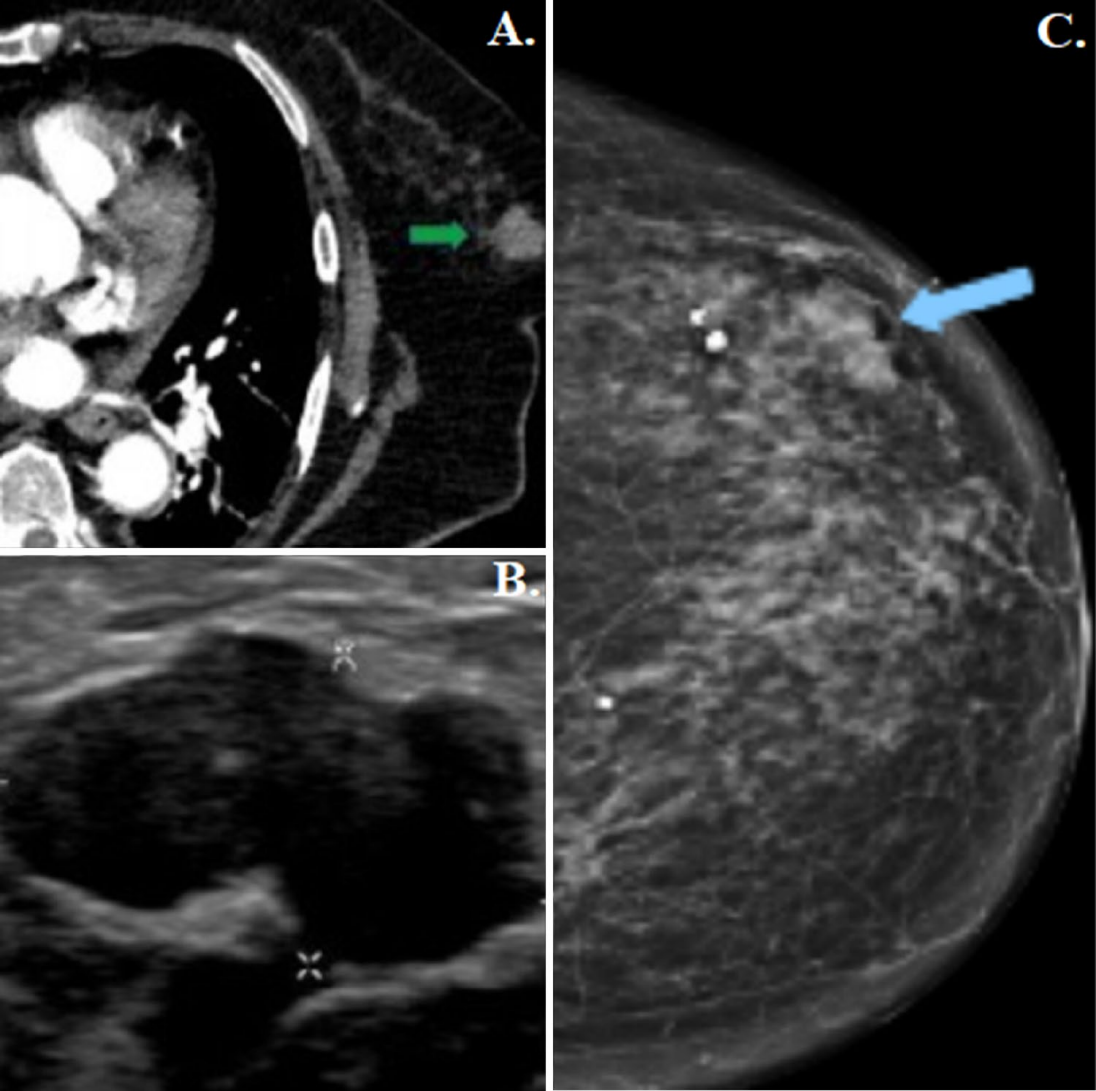
Fibroadenoma. **A** Left breast tissue CT imaging after organic iodinated contrasted agent intravenous injection presenting a nodular tumour with well-defined and lobulated margins lacking marked contrast enhancement (green arrow). **B** Mammary ultrasound showing a hypoechoic tumour with sharp and polylobed margins. **C** Mammogram in cranio-caudal projection showing a homogenous mammary thickening at the junction of the left breast outer quadrants (blue arrow)

A bioptic assessment was required in 43 (63.3%) patients, confirming the suspected malignancy in 35 (81.4%) cases, and rectifying it in 2 (4.6%) (Fig. 1). A breast radiologist deemed the biopsy unnecessary in the remaining 25 (36.7%) cases included in the study, as their nodules already clearly presented mammographic and ultrasound features of benignity.

### Correlation between CT characteristics, BI-RADS score and histopathological report

**Fig. 2:**
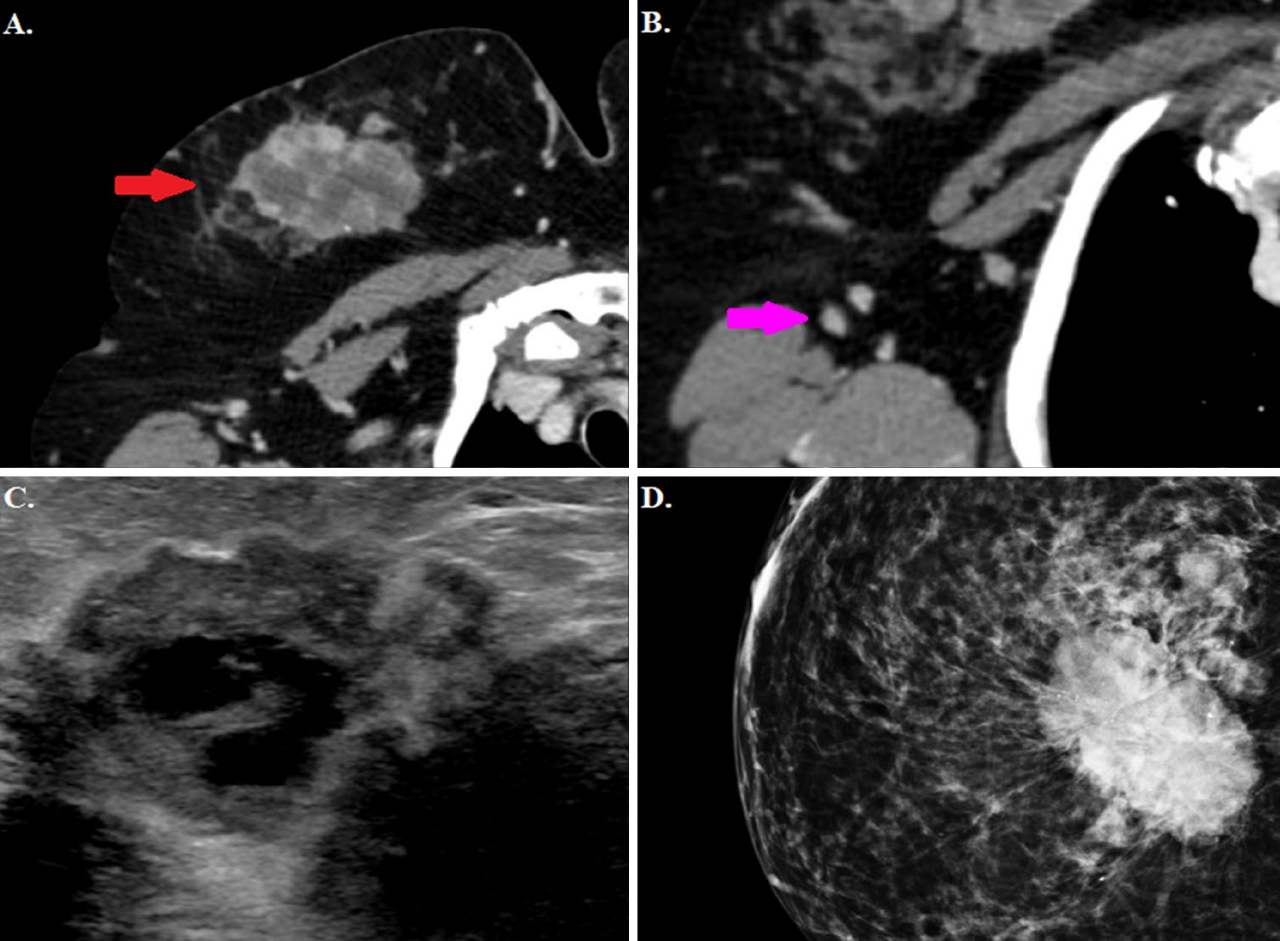
Invasive ductal carcinoma. **A** Breast tissue CT imaging after intravenous injection of organic iodinated contrast agent showing a nodular tumour with undefined margins and a marked and inhomogeneous contrast enhancement (red arrow); **B** Homolateral lymph node CT imaging appearing increased in size, with marked contrast enhancement and lacking a clearly recognisable hilum (pink arrow). **C** Mammary ultrasound with a highly hypoechoic nodule with irregular margins and acoustic shadow. **D** Mammogram showing a thickened asymmetric breast tissue with irregular margins and intramodular microcalcifications

**Fig. 3:**
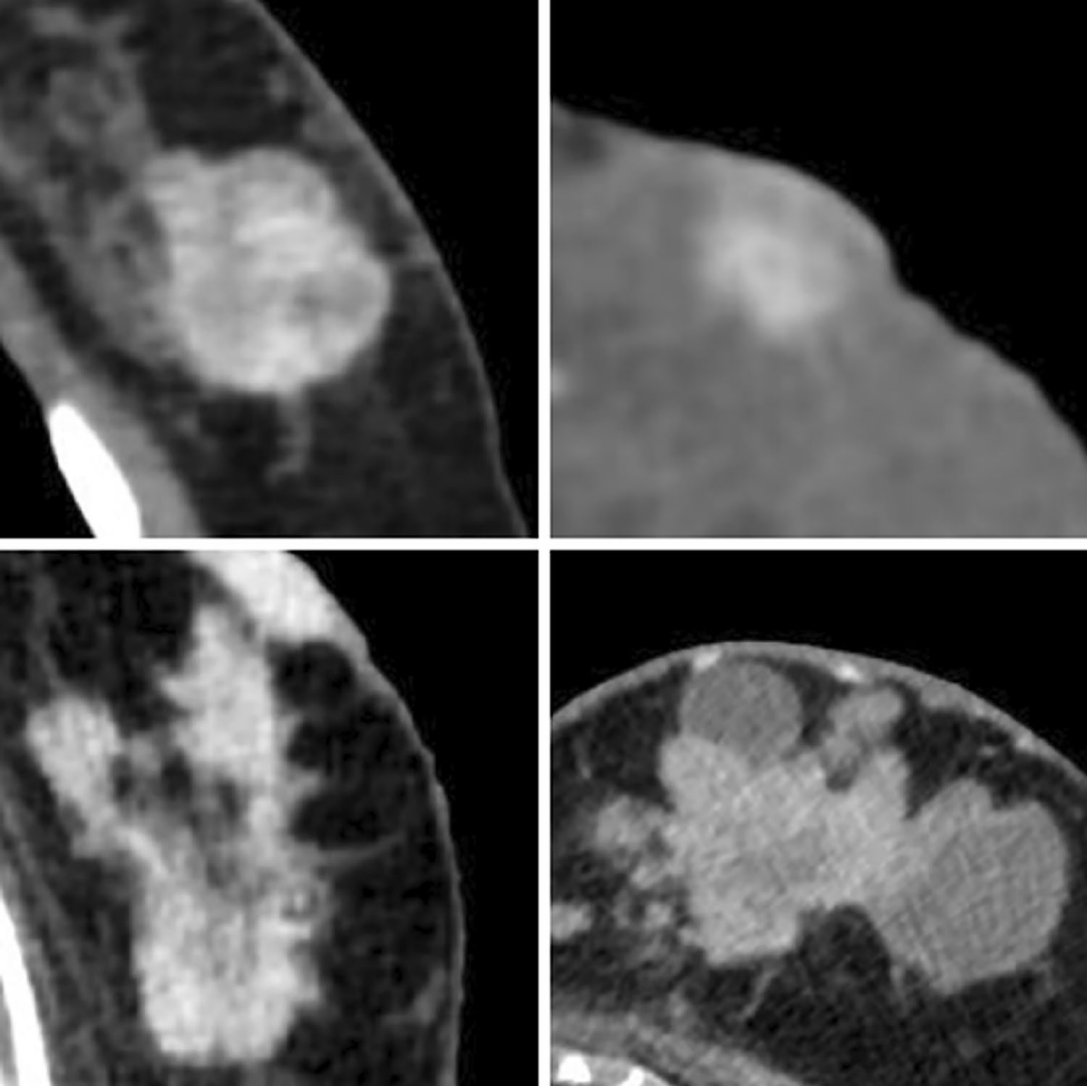
Post-contrast enhancement

**Fig. 4:**
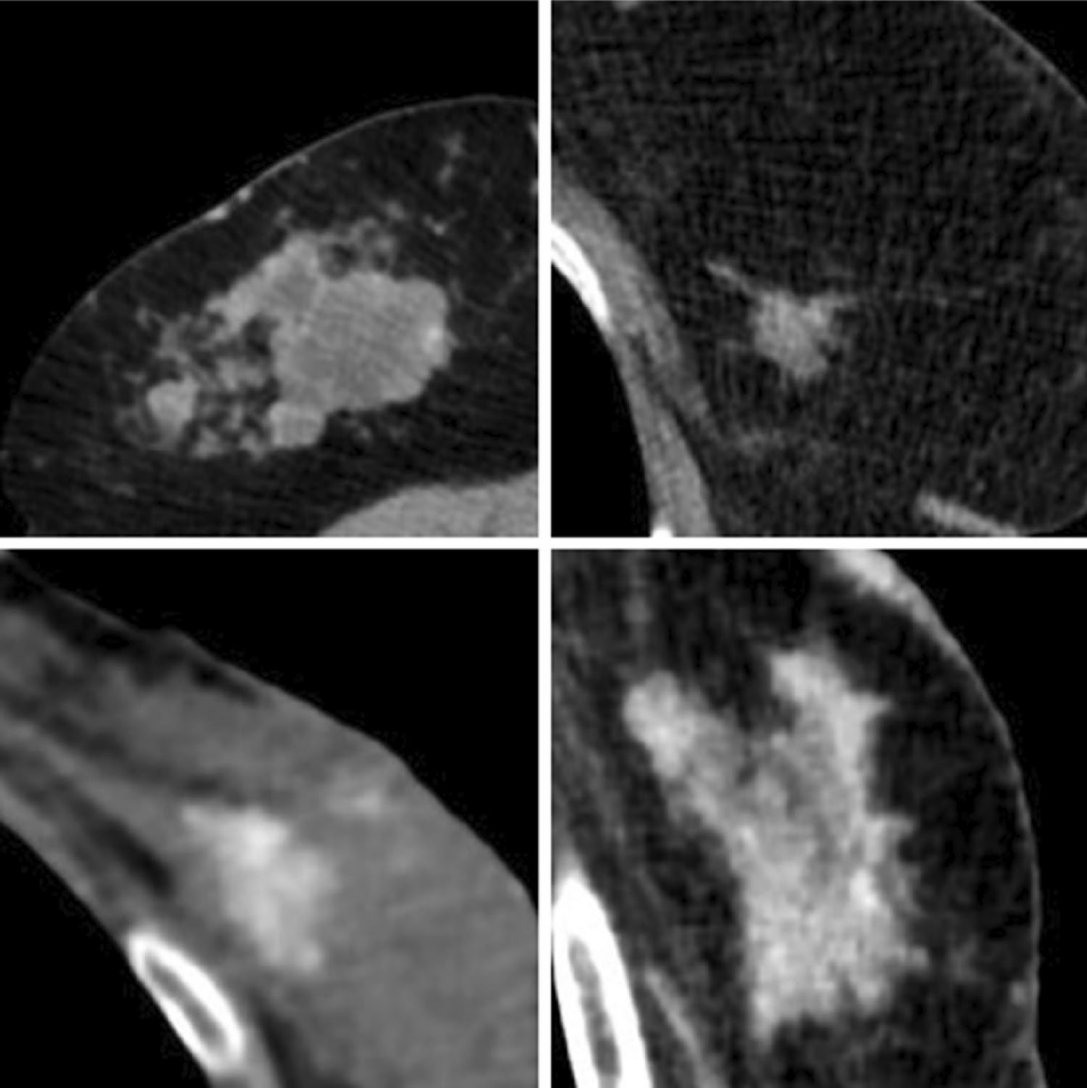
Margin irregularity

**Fig. 5:**
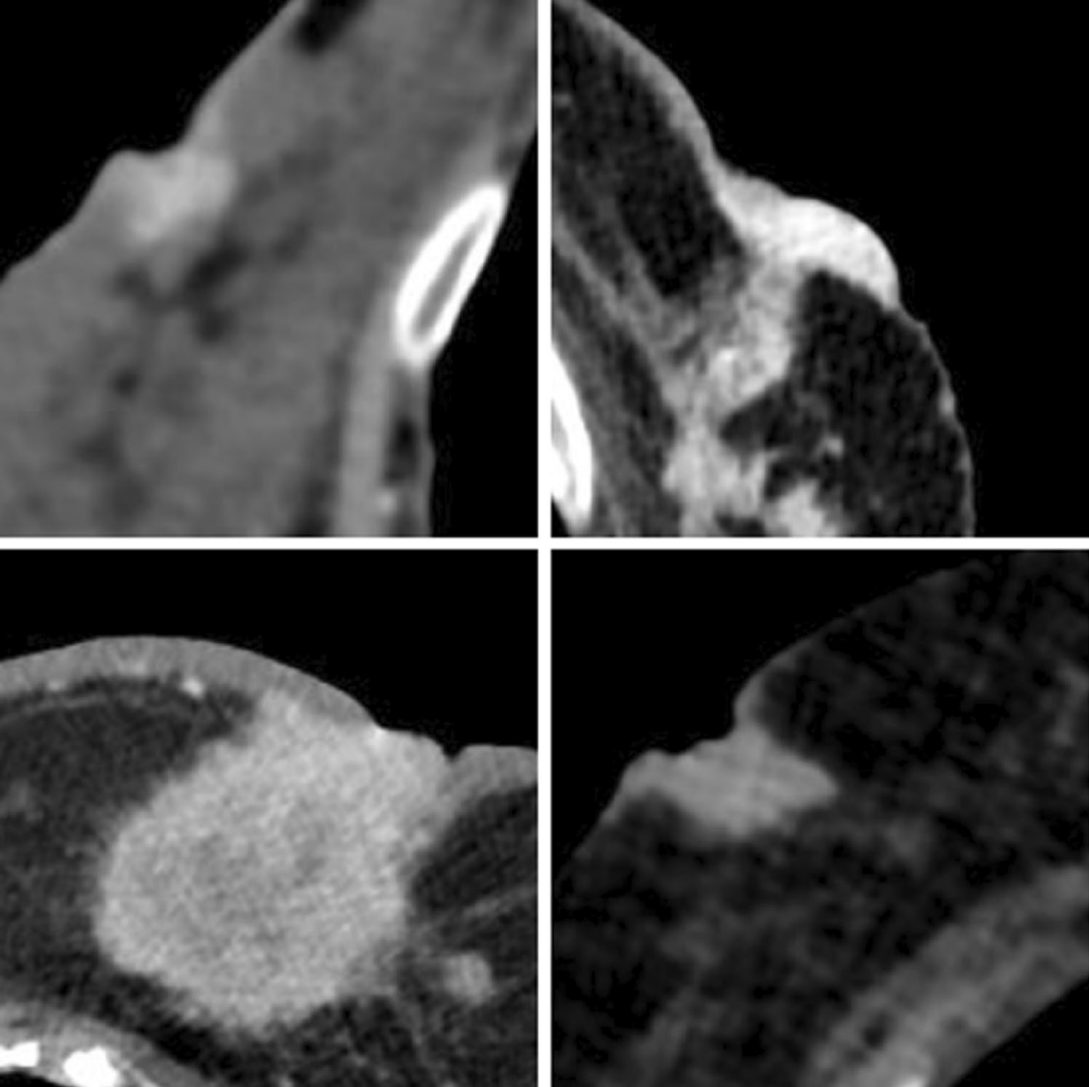
Nipple retraction

**Fig. 6:**
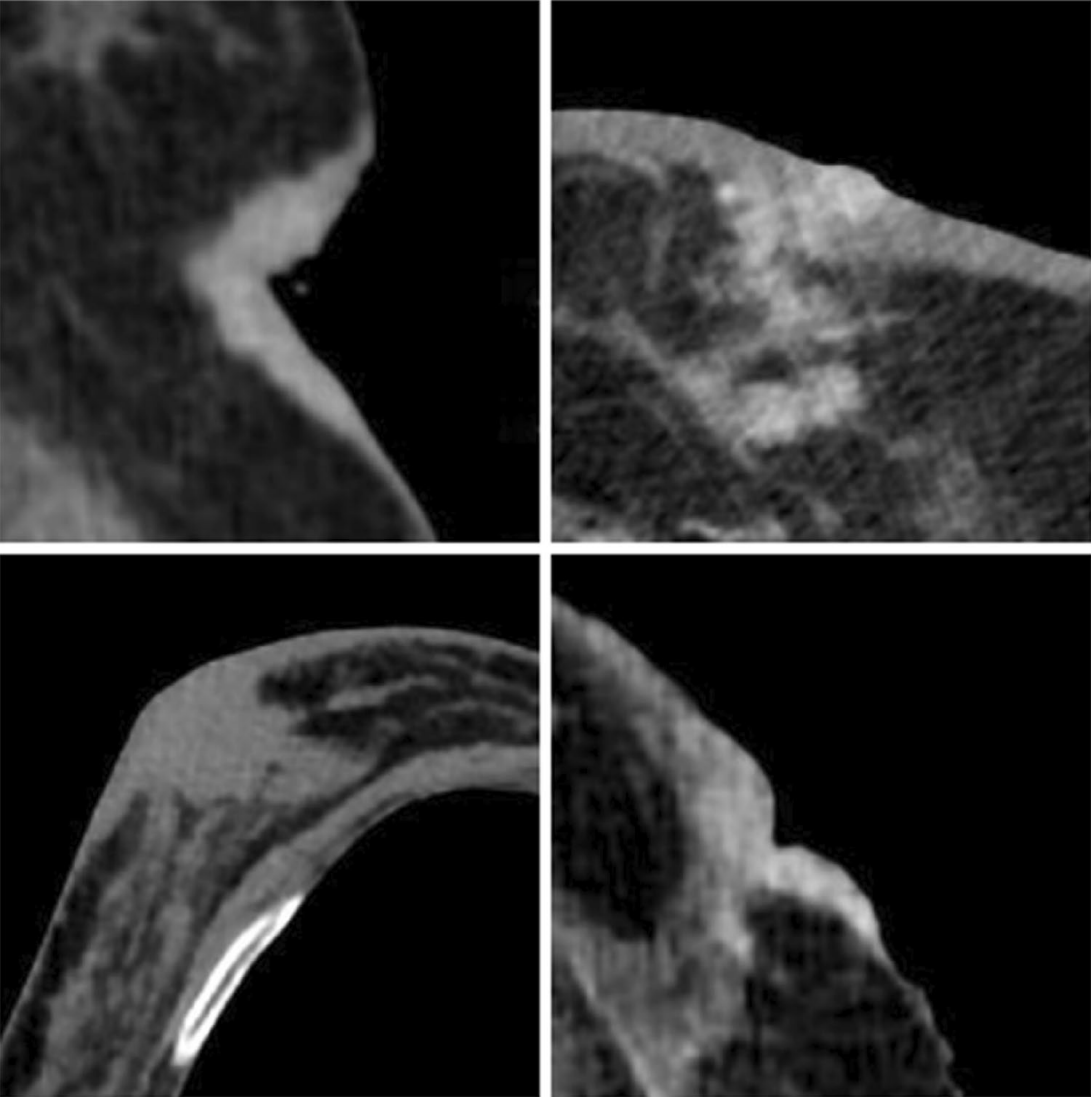
Skin thickening

**Fig. 7:**
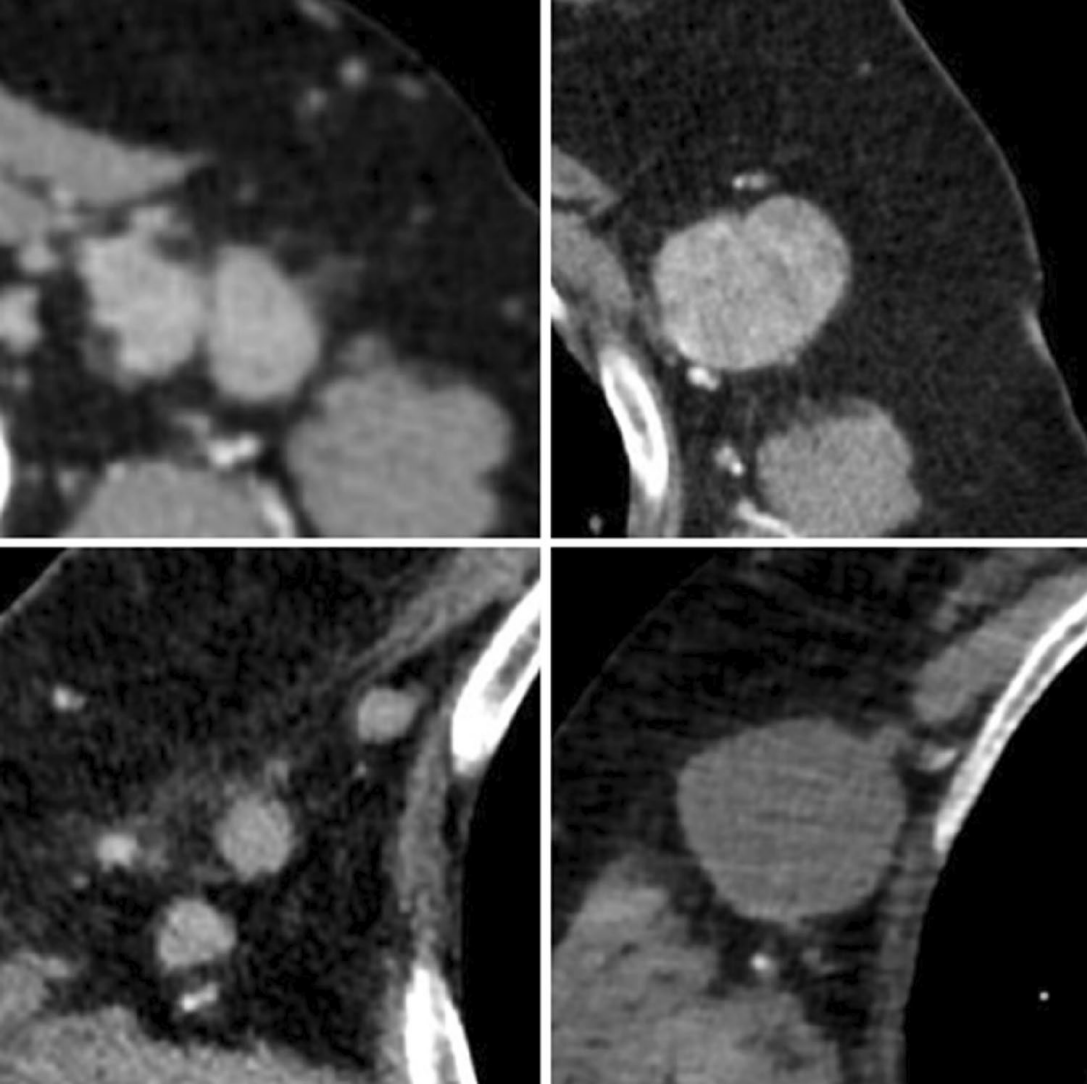
Atypical lymph nodes, suspicious for metastatic involvement

Pearson’s Chi-square test was used to analyse the correlation between the nodule CT characteristics and the likelihood of obtaining a BI-RADS 5 score on mammographic evaluation (Fig. 2). The CT findings most likely to determine a BI-RADS 5 on a mammogram were post-contrast enhancement (*p* = 0.001) (see Fig. 3), margin irregularity (*p* = 0.0001) (see Fig. 4), nipple retraction (*p* = 0.001) (see Fig. 5), skin thickening (*p* = 0.024) (see Fig. 6), and the presence of structurally atypical lymph nodes suspicious for metastatic involvement (*p* = 0.0001) (see Fig. 7). The CT features most likely predictive of a biopsy positive for malignancy were post-contrast enhancement (*p* = 0.0001), margin irregularity (*p* = 0.0001), and the presence of suspicious lymph nodes (*p* = 0.011) (Table 2).

**Table 2:** Correlation between CT nodule characteristics, BI-RADS 5 scores, and histopathological findings

CT characteristics	Focality	Post-contrast enhancement	Margins	Microcalcifications	Nipple retraction	Skin thickening	Lymph nodes
BI-RADS 5	2	1	1	2	1	1	1
Histopathological report	2	1	1	2	2	2	1

1 = statistically significant correlation, *p* ≤ 0.05

2 = no correlation

All the CT characteristics suspicious for malignancy are summed up in Table 3 with iconographic correlation.

**Table 3:** CT characteristics suspicious for malignancy with iconographic correlation

CT characteristics suspicious for malignancy
Post-contrast enhancement: see Fig. 3
Margin irregularity: see Fig. 4
Nipple retraction: see Fig. 5
Skin thickening: see Fig. 6
Atypical lymph nodes: see Fig. 7

Considering enhanced and unenhanced CT, the correlation between benign and malignant nodules and CT was not statistically significant (*p* = 0.106).

### Correlation between nodule malignancy and size

The lesions’ size was divided into quartiles (Table 4). An OR statistical analysis showed that a 1.13-mm increase in size of the tumour was associated with a greater risk of a BI-RADS 5 finding on a mammogram, while a 1.11-mm growth was associated with a greater risk of malignancy evidenced by histopathological examination.

**Table 4:** Lesions’ size divided into quartiles with bioptic results correlation

Size (mm)	Malignancy	Benignity	Biopsy unnecessary	Total
0–10	6	3	8	17
11–15	5	4	9	18
16–24	8	1	7	16
25+	16	0	1	17
total	35	8	25	68

## Discussion

Between January 2016 and April 2022, the Radiology Department at AOU Maggiore della Carità detected 158 mammary lesions as incidental findings on chest CT: 68 patients were followed up in our centre. Upon histological examination, 35 of these patients were diagnosed with breast cancer. Even though breast cancer is notoriously the most frequent cancer type among women [1, 2], in our series of 68 patients we found 3 rare cases of male breast cancer. Besides gender, age is a well-known risk factor for breast cancer development: the incidence rate is lower under 40 years old, while it is higher over the age of 70. In our study, we recorded a mean age at the time of diagnosis of 66.2 years in women and 72.3 years in men.

Another potentially interesting observation of our study is that 63.4% of patients with a working diagnosis of cancer developed a malignant breast tumour, whereas only 42.1% of patients with a working diagnosis unrelated to cancer had a final diagnosis of breast cancer. Although due to the limited sample size, we could not achieve statistical significance (*p* = 0.08) by Chi-square test with Yates’ correction, these findings suggest an increased risk of multiple primary cancer in patients with a working diagnosis of cancer. In good agreement, a large retrospective study by Tarjak et al. showed that, out of 109,054 cancer patients, 1.63% developed a synchronous or metachronous malignancy within 2 months from the primary tumour [13]. The analysis of the concordance between the primary site and the synchronous or metachronous tumour incidence rate led the authors to conclude that breast cancer is the most frequent multiple cancer, especially when it coexists with genetic syndromes, such as breast cancer gene 1 and 2 (BRCA1 and BRCA2) gene mutations, Li-Fraumeni syndrome, hereditary diffuse gastric cancer, Peutz–Jeghers syndrome and PTEN hamartoma tumour syndrome [14, 15].

Among the CT signs indicative of malignancy is post-contrast enhancement, which is predictive of both BIRADS-5 on subsequent mammography and cancer diagnosis on histopathological examination [16–18]. A further contribution can be given by the morphological analysis: the irregularities of the lesion margins were found to be predictive of both possible BI-RADS 5 classification on mammography and malignancy on histopathological examination [17, 19]. The presence of structurally atypical axillary lymph nodes (shorter axis < 1 cm, rounded in shape, loss of the fatty hilum) was also found to be indicative of a possible malignancy [18, 19].

Finally, we compared the nodule characteristics on CT that we found to be predictive of breast cancer with those reported in the literature. From this comparison, it emerges that our results are in good agreement with those published by Lin et al. [17] and Georgieva et al. [18], while they differ from the findings by Prionas et al. [16] and Moyle et al. [19] (see Table 5). One of the major limitations to these studies lies in their retrospectivity, which also flaws our findings. Thus, a multivariate analysis of CT characteristics on a wider sample size is warranted.

**Table 5:** CT characteristics suspicious for malignancy in our study *vs* those reported in the existing literature

CT characteristics	Post-contrast enhancement	Margin	Lymph nodes
Our study (68 patients)	1	1	1
Prionas et al. (46 patients)	1	3	3
Lin et al. (97 patients)	1	1	1
Georgieva et al. (27 patients)	1	1	2
Moyle et al. (70 patients)	2	1	2

1 = significant for malignancy

2 = non-significant for malignancy

3 = not mentioned

## Conclusions

Incidental breast lesions can often be identified on chest CT scans. Although CT does not allow to properly classify a tumour, the evaluation and description of some of its characteristics may help hypothesise a diagnostic suspicion of benignity or malignancy.

Our data, in keeping with the existing literature, suggest that the incidental detection of a mammary nodule on CT imaging should raise the suspicion of malignant breast cancer in the presence of post-contrast enhancement, irregular margins, and structurally atypical lymph nodes (shorter axis < 1 cm, rounded in shape and loss of the fatty hilum). Furthermore, the likelihood of malignancy appears to be enhanced in patients with a working diagnosis of cancer. Conversely, the absence of these semiological findings appears to be, most of the times, associated with benign tumours. Hence, in case of incidental finding of breast lesions, the careful evaluation of the CT parameters described in our study can guide the radiologist towards the correct diagnosis of benign or malignant breast pathology.
